# Anticipated Effects of Using a Poststroke Patient Portal for Stroke Survivors and Carers: Semistructured Interview Study

**DOI:** 10.2196/89787

**Published:** 2026-11-11

**Authors:** Sophie Rowland-Coomber, Zhiqiang Huo, Mahnoor Mustafa, Eleanor Stevens, Christopher McKevitt, Timothy Neate, Martin Chapman, Matthew DL O'Connell, Eva S Emmett, Ajay Bhalla, Vasa Curcin, Charles D A Wolfe, Iain J Marshall, David Wyatt

**Affiliations:** 1 Department of Primary Care & Data Science, School of Life Course and Population Health Science, King's College London, Addison House, Guy's Campus, London, England, SE1 1UL, United Kingdom, 020 7188 7188; 2 Wolfson Institute of Population Health, Queen Mary University of London, London, England, United Kingdom; 3 Department of Language and Communication Science, School of Health & Psychological Sciences, City St George's, University of London, London, England, United Kingdom; 4 Department of Informatics, King's College London, London, England, United Kingdom; 5 Department of Ageing, Health, and Stroke, Guy’s and St Thomas’ National Health Service Foundation Trust, London, UK, London, Middlesex, United Kingdom; 6 NIHR Applied Research Collaboration (ARC) South London at King’s College Hospital NHS Foundation Trust, UK, London, Middlesex, United Kingdom; 7 Department of Society and Politics, Aston University, Birmingham, England, United Kingdom

**Keywords:** stroke, patient portal, qualitative research, caregivers

## Abstract

**Background:**

Digital technologies are increasingly incorporated into long-term self-management of health conditions, yet their social impact on stroke survivors and carers remains limited.

**Objective:**

This study explores how stroke survivors and carers view a prototype web-based application (“portal”) that allows patients to access and contribute up-to-date information about their health and care poststroke and how this might impact their self-management practices.

**Methods:**

Through 27 semistructured interviews with 21 stroke survivors and 6 informal carers (performing unpaid care work), we explore the enablers of and barriers to the adoption and use of a portal for stroke survivors. To support discussions, participants were shown images of the portal prototype that displayed various features, including a self-living ability assessment, blood pressure monitoring, and an overview of exemplar personal data.

**Results:**

Four themes were identified to articulate the anticipated effects of the portal: (1) portals as potential tools to empower through enabling survivors and carers to access stroke knowledge and enhance self-management; (2) portals as a method that could offer practical support for recovery management, therefore helping survivors manage their recovery; (3) hesitancy about using a portal, particularly regarding tracking recovery through personal data input; and (4) portals as a method to potentially share information to help keep family, friends, and wider support networks informed about their health and its management. Anticipated experiences also varied by social position. Young stroke survivors, for example, said they would value age-appropriate peer connections, while carers anticipated concerns about additional workload. Participants also felt that family members often lacked understanding of stroke impacts, suggesting that portals could help facilitate better communication about invisible and complex disabilities.

**Conclusions:**

The anticipated benefits of digital tools for empowering patients need to be weighed against the complexity of people’s health and the additional responsibility digital interventions place on carers to communicate for those with severe disabilities. Our findings suggest that for a portal to be effective, it would need to address not only people’s medical needs but also their social needs, especially their use of informal care. Further research is needed, including with an implemented portal, to understand the use of portals over extended periods.

## Introduction

Stroke is a leading cause of long-term disability in the UK, and its prevalence is estimated to grow by 120% between 2015 and 2035 [1]. While acute care has improved significantly, stroke survivors face well-documented challenges in the postdischarge period: navigating fragmented systems of care, managing secondary prevention, and coping with the gradual withdrawal of formal support [2-4]. These challenges become particularly acute from around 6 months poststroke, when survivors are largely left to self-manage their recovery, often relying heavily on informal carers [5,6].

Digital health interventions are increasingly positioned to support this long-term self-management. The National Health Service Long Term Plan, for example, envisages a growing role for digital tools in supporting people to manage their conditions at home while remaining connected to effective rehabilitation [7]. The need for the delivery of effective, timely, and positive outcomes within health care is essential. Studies on digital health interventions have spanned areas of illness such as diabetes [8,9], asthma [10,11], arthritis [12], high blood pressure [13], and stroke [14]. Despite their differences, these studies have all focused on improving outcomes through the provision of information to professionals and/or the public. This illustrates the increasing role that digital health interventions are expected to play in enabling people to feel better informed in relation to their health [15].

Patient portals, secure web-based applications through which people can access their health information and contribute their own data, are expected to play a key role in this vision [3]. Existing evidence suggests that when they are well-designed, portals can support engagement with health information, facilitate condition monitoring, and enable more informed decision-making [16]. Lenihan and Smallson [16]identify three conditions for meaningful portal engagement: access to knowledge about disease and care, support for becoming better connected to and educated about one’s condition, and a reduction of the barriers to digital literacy and access.

Yet the design of portals has tended to foreground concerns about the patient-provider relationship [17], the integration of multiple information types to support specific rehabilitation pathways [18], and educational content aimed at reducing risk [19]. There has been comparatively less attention given to what stroke survivors and carers themselves expect from such tools. Portals designed without sustained attention to user expectations risk reproducing the very disconnect from formal care that they are meant to address. Similarly, while portals are often framed as instruments of health equity [20], they may equally entrench inequalities where underlying concerns around digital access, data sharing, and security are not engaged with at the design stage [21]. Understanding what survivors and carers expect of a portal practically, functionally, and in terms of its place in their wider experience of care is therefore an important and necessary step in design.

This paper reports on a qualitative study conducted as part of the development of a prototype patient portal linked to the South London Stroke Register (SLSR), a long-running observational cohort study of stroke incidence and outcomes, which collects data from participants at different time points from 48 hours poststroke [22]. The SLSR and portal form part of a wider program of work exploring how health data can be used to improve the lives of stroke survivors [23-25]. The portal, drawing on SLSR data and data input by stroke survivors and carers, aims to provide a platform for stroke survivors to interact with their data to support self-management and improve health outcomes. This qualitative study aimed to explore stroke survivors’ and carers’ expectations for such a portal, examining their views on practical design, function, and features, alongside the wider role they see for digital technologies in stroke management.

## Methods

### Overview

Participants were recruited to this study through stroke support charities and the SLSR [22]. In examining the role of the patient portal, we conducted 27 semistructured interviews with 21 stroke survivors and 6 informal carers. Images of the portal prototype were incorporated into the interviews to solicit participants’ views about the portal. Visual representation of the portal was considered the most effective mechanism for the initial perspectives on how they might choose to interact with the portal, with an interactive prototype of the portal subsequently made available for a usability study [14].

In the interview, participants were shown early iterations of the patient portal in the form of screenshots from the login screen (Figure 1), patient dashboard (Figure 2), and self-living ability assessment (Figure 3). The portal prototype displayed health information and data relating to a fictional user, Daniel, a 56-year-old man. During the interviews, we explained how the portal was intended to function, using images to illustrate its various features (eg, self-living ability, well-being assessment). We also discussed with participants that while there was no direct linkage between the SLSR and general practitioner or hospital records, they would, through the portal, have access to information collected routinely as part of the SLSR follow-up; for example, the following were incorporated into the portal through a section called Health Records: medication, Barthel Index (a measure of disability level), Frenchay Activities Index (a measure of activity level), and the 12-item Short-Form survey (SF-12), a measure of health-related quality of life.

**Figure 1.**
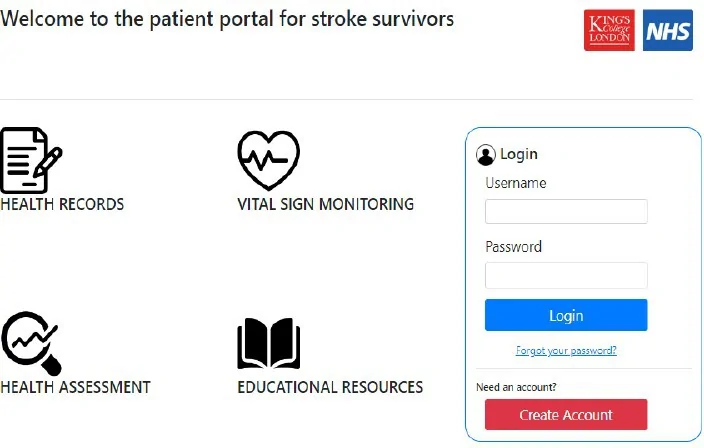
Patient portal login page.

**Figure 2.**
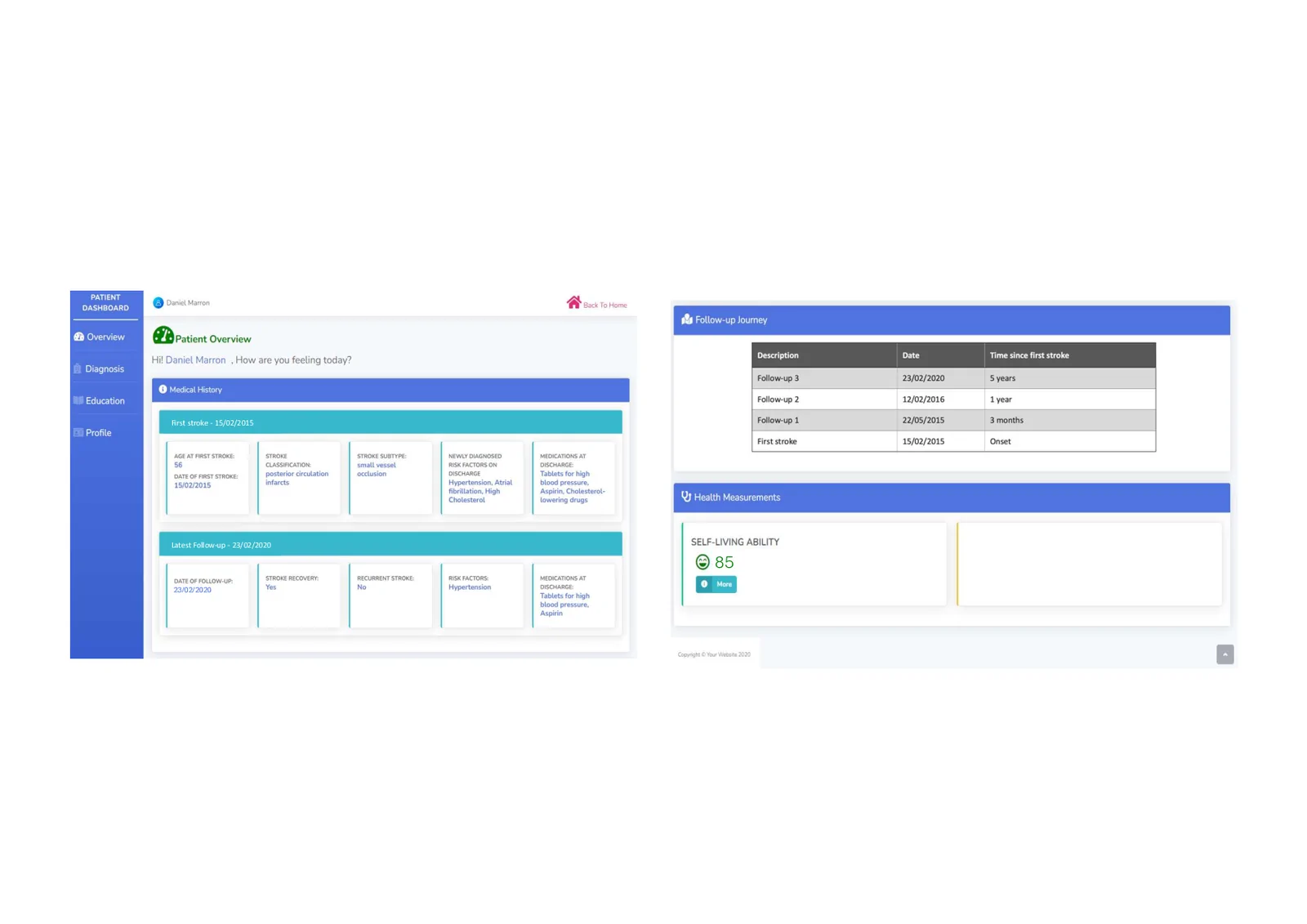
Patient overview page.

**Figure 3.**
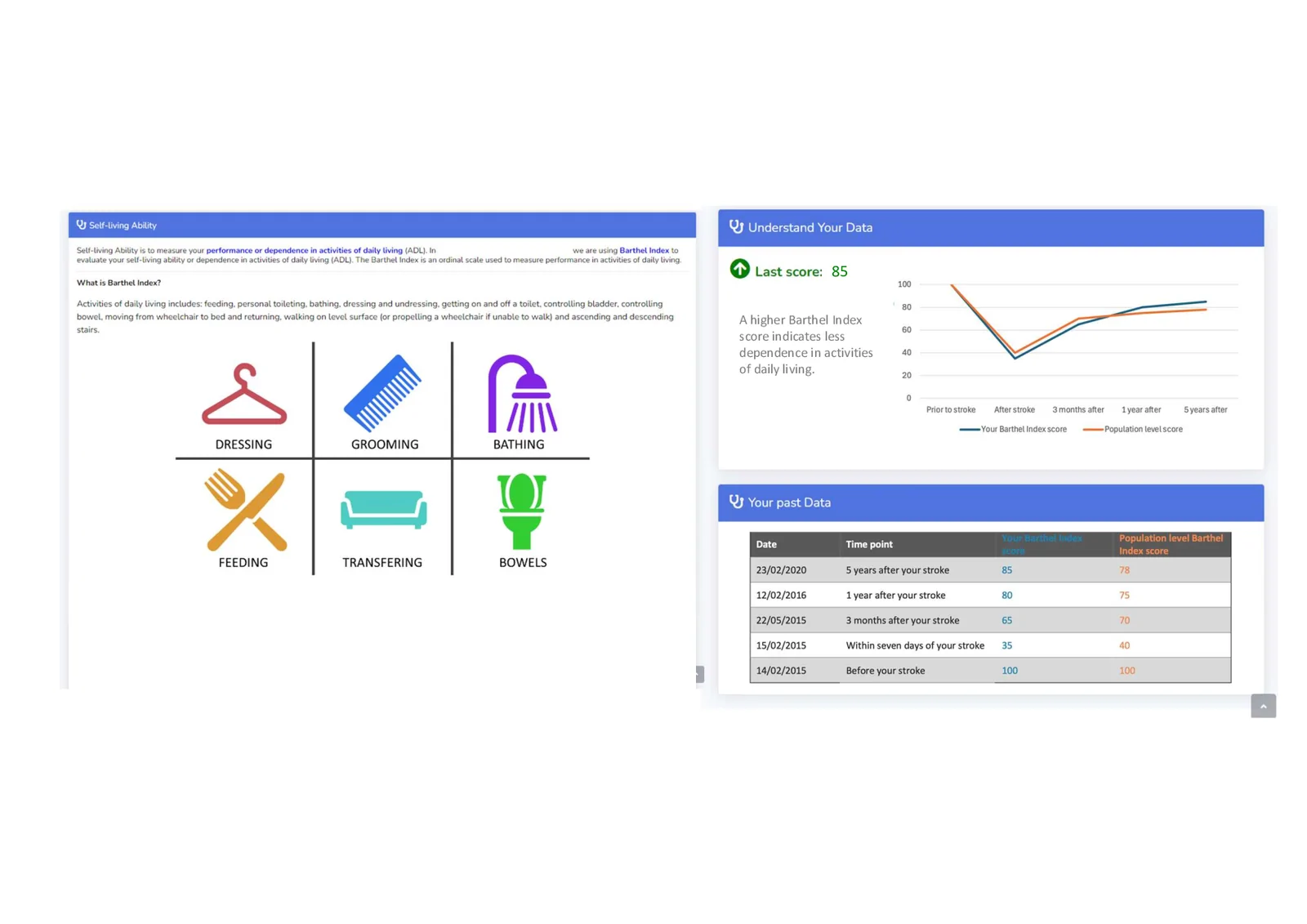
Self-living ability assessment page.

At the start of the interview, it was made clear to participants that health care providers would not routinely have access to the data entered in the portal. However, this did emerge within the interviews when discussing education features and the expansion and/or improvement of the prototype portal in the future (see Multimedia Appendices 1 and 2 for a list of features envisioned for the portal). During the interview, participants were asked to reflect on the portal’s design, the relevance of the data being collected and displayed to them personally, and the perceived barriers to engagement. Engaging participants through photographic images fostered an environment that enabled a “holistic” and “multifaceted” understanding, creating meaningful discussion [26].

Reflexive thematic analysis was conducted to account for limitations within the study and to provide clarity on the construction of themes [27]. The research team identified themes about the role digital technologies, such as a portal, can have in assisting and/or enhancing stroke care and recovery. One author (SR-C) read transcripts and conducted initial coding using NVivo 14 (version 14; Lumivero). Initial coding saw the data split into themes; these were then shared with DW and MM to further refine the codes. Once these three authors had met and discussed them, the codes were then shared with a wider team for discussion both analytically and in terms of tangible changes that could be incorporated into the portal design. This enabled the authors to consider how digital technologies could impact physical and emotional recovery and how the portal functioned, alongside raising wider sociological questions about the role digital technologies can play in health management and care.

### Ethical Considerations

Ethical consent for this study was obtained through the Health Research Authority and Health and Care Research Wales (research ethics committee reference number 21/WS/0145).  Interviews took place online via Microsoft Teams to ensure that participants were recruited and could share representative stroke experiences in England and Wales. In-person interviews were offered to participants living within the London area or close to research team members. Written consent was obtained from participants before the interview. In accordance with feedback from the project’s Patient and Public Involvement group (PPI), interviews lasted on average between 30 and 40 minutes to avoid participants becoming overly fatigued. All interviews were recorded and transcribed.

## Results

Participants were from diverse ethnic backgrounds and ages; the youngest stroke survivor was aged 31 years and the oldest was aged 88 years. Participants were at different time points in their recovery, with the length of time between stroke and interview ranging from a few months to 17 years (see Tables 1 and 2). The research team identified key themes relating to “portal design,” “managing health poststroke,” and “health information,” which after further refinement resulted in the identification of four themes relating to “empowerment,” “assistance,” “hesitancy,” and “community.” Participants considered the potential role that the portal might play in creating a positive environment if they were to have access to medical knowledge, a connection to other stroke survivors, and support in recovery, as well as when they needed to understand when to seek help for chronic illness. Despite many participants recognizing the benefits that a portal could offer to their understanding of stroke, some participants admitted to being hesitant about using a portal, being unclear what it would add to their stroke recovery, and feeling hesitant about the tensions this might create between the burden of their health condition and the additional burden (and potential benefits) of a digital intervention [28]. In examining the role of digital technologies in the management of chronic illness and its implications for stroke, we present our findings through 4 distinct but interconnected themes.

**Table 1.** Summary of participant demographic characteristics.

Participant characteristics	Participants (N=27), n (%)
Status
Stroke survivor	22 (79)
Carer	6 (21)
Gender	
Male	14 (50)
Female	14 (50)
Age at time of stroke, years	
<50	2 (7)
50 to 65	14 (50)
66 to 79	6 (21)
>80	3 (11)
Unknown	3 (11)
Ethnicity*	
White	16 (57)
Black	4 (14)
Asian	2 (7)
Arab	1 (4)
Unknown	5 (18)
Year of stroke	
2009 or earlier	2 (9)
2010 to 2015	5 (22)
2016 to 2019	3 (14)
2020 or later	7 (32)
Unknown	5 (23)
Location in the UK	
Southeast (including London)	21 (75)
South	2 (7.1)
South Midlands	1 (3.6)
Northeast	2 (7.1)
Northwest	1 (3.6)
Unknown	1 (3.6)
Recruitment	
South London Stroke Register	7 (25)
Stroke charity	21 (75)

**Table 2.** Summary of individual participant characteristics.

Participant pseudonyms	Status	Year of stroke	Gender	Age	Ethnic group	Recruitment method	Location in UK
Elisabeth	Stroke survivor	2011	Female	50-65	White	Stroke charity	Southeast
Jacob	Stroke survivor	2010	Male	50-65	White	Stroke charity	Northwest
Emma	Carer	2018	Female	50-65 (>80^a^)	White	Stroke charity	London
Samuel	Stroke survivor	2020	Male	50-65	White	Stroke charity	Northeast
Shirley	Stroke survivor	2016	Female	>80	—^b^	Stroke charity	London
John	Carer	2021	Male	66-79 (>50‐65^a^)	White	Stroke charity	Northeast
David	Stroke survivor	—	Male	66-79	White	Stroke charity	London
Caroline	Stroke survivor	2023	Female	50-65	White	Stroke charity	South
Robert	Stroke survivor	—	Male	>80	White	Stroke charity	London
Hannah	Stroke survivor	2021	Female	50-65	White	Stroke charity	—
Georgina	Stroke survivor	2022	Female	50-65	White	Stroke charity	South
Miriam	Stroke survivor	2021	Female	<50	Asian	SLSR^c^	London
Maria	Stroke survivor	2006	Female	66-79	Asian	SLSR	London
Julia and Liam	Julia: carer Liam: stroke survivor	2018	Julia: femaleLiam: male	Julia: — Liam: 50-65	Julia: White Liam: White	Stroke charity	Southeast
Eleanor	Stroke survivor	2011	Female	66	Black	SLSR	London
Sarah	Carer	2021	Female	50-65	White	SLSR	London
Ali	Stroke survivor	2009	Male	66-79	Arab	SLSR	London
Matthew	Stroke survivor	2010	Male	50-65	White	Stroke charity	South Midlands
James	Stroke survivor	—	Male	50-65	White	Stroke charity	London
George	Stroke survivor	2017	Male	>80	—	Stroke charity	London
Thomas	Stroke survivor	—	Male	—	—	Stroke charity	London
Gemma and Chris	Gemma: carer Chris: stroke survivor	2020	Gemma: femaleChris: male	Gemma: 66-79 Chris: 50-65	Gemma: Black Chris: White	Stroke charity	London
Amelia and Adam	Amelia: carer Adam: stroke survivor	—	Amelia: femaleChris: male	Amelia: 50-65 Chris: 66-79	Amelia: Black Adam: —	Stroke charity	London
Theresa	Stroke survivor	2010	Female	—	—	SLSR	London
Jack	Stroke survivor	2021	Male	<50	Black	SLSR	London

a Number in parentheses indicates the age of the stroke survivor cared for by the participant at the time of the interview.

b Information not available.

c SLSR: South London Stroke Register.

### Empowerment: Improving Knowledge of Stroke

During the discussion of the portal prototype, nearly all participants made clear their eagerness to establish an environment in which they felt empowered. To achieve this, stroke survivors and carers required knowledge of stroke, which would allow them to better manage their health in their everyday life. For many participants, the portal played an important role in communicating risk associated with stroke, such as healthy ranges for blood pressure, and when they need to do something:

I was...discharged and just told, “If your symptoms get worse, you need to come back.” But nobody ever sort of defined...and I know it’s different for everybody, but nobody ever said to me, “If you do this, it’s going to cause extra fatigue, you don’t need to worry about it. If you start to feel like this, that’s not something you need to worry about.” So, I guess what I wanted was something that said “These are the symptoms you need to be really worried about and come straight back with,” as opposed to “I’m really feeling really rough today, but if I lie down for a couple of hours, I’ll probably be okay” type thing.

[Hannah, stroke survivor]

Like many participants, Hannah reflected on the expectation that she should self-manage her stroke recovery following hospital discharge. Responsibility is placed on stroke survivors to know when and if to seek help, which can cause anxiety and worry. Identifying credible sources of information is a key part of managing this worry and supporting stroke recovery. Digital tools can encourage people to think positively about changes in their health, especially when they know they are receiving information from a reputable source. In several interviews, discussion of the portal prototype prompted stroke survivors and carers to acknowledge the changes that the stroke survivors’ bodies had undergone, and participants said that the portal could offer a foundation on which to manage and plan for long-term recovery:

I think the medical records [included in the portal] can show the truth about what the issues are. Because then it helps you to understand, “Why can’t I do this thing?” Losing your cerebellum shouldn’t prevent you from doing this thing because it’s about balance and movement....In the early days, you sort of fall into a bit of a pit, and you don’t really think you can do anything, and then slowly and slowly you realize you can do much more than you thought, and then with more and more practice and exercise and stuff, you can do it.

[Miriam, stroke survivor]

The portal acted as an intermediary between stroke survivors and carers. Through nonintrusive and supportive of self-management practices, the portal could enable stroke survivors to manage their recovery at a pace more in line with their physical and mental preparedness. Participants indicated that portals should offer support and serve as an extension of health care service provision, thereby creating confidence in self-management of stroke through knowledge and guidance.

### Assistance: Managing Stroke Recovery

Participants described the challenges that individuals experience in navigating the health care system. When asked during the interview, many appeared unclear regarding the resources and opportunities for support available to them. In learning to adjust to their physical impairment, several participants noted that they had become detached from their prior fitness routines and, as such, were no longer fit and were disengaged from recovery. Therefore, they argued that the potential portal offered motivation to engage in recovery:

When you’ve had a stroke, you’re somewhere halfway between being alive and dead. I don’t know. I don’t know what the purpose is anymore sometimes...I think it will help get us motivated...just keeping the record there, even if I don’t do anything more than just opening it up every day, putting all the stuff in it that I need to and then closing it down.

[James, stroke survivor]

Participants noted the benefit of the portal in enabling people to stay informed in managing their health and therefore to gain control over their condition. Many were keen to stress how a portal could assist them and those around them in this process. The portal could serve not just as a site of information but as a tool in facilitating self-management. Participants highlighted the potential of a portal to improve well-being by enabling stroke survivors to track progress and recovery more broadly using the self-living ability page (Figure 3):

I think if you are tracking progress and stuff on there, that would be a real good benefit to your mental health. I know a lot of people complain about, “Oh I’m not getting anywhere. I feel like nothing is changing, nothing’s improving.” And I quite often say to people, “Keep a diary of what you’ve done, so if today you’ve walked 10 steps, write it down, and if tomorrow it’s 11...but when you look back you can see you’ve done a little bit more.” So, something like that I think is a boost to morale.

[Hannah, stroke survivor]

Recovery is complex, yet the potential to monitor achievements through a portal would help ease several participants’ frustration and tension that arose through their inability to complete tasks they performed easily prior to their stroke. In addressing the invisible effects of stroke (eg, fatigue, emotional lability, depression, anxiety, and cognitive impairment) through monitoring and assessment of health changes, accessing the portal could provide a quick insight or overview of an individual’s health:

Having the timeline, I think, from the patient perspective is important. For a medical record perspective, you want other things prioritized. For a patient, you want to know...and medical records now don’t have this, they don’t...it used to be there’d be a book, so you just flick through the book, and you know it’s at the front or the back depending on the way it’s done. And there should be a summary page which is what this is. So just one page only—one screen, one page—that has essential demographics and the timeline and the drugs.

[Gemma, carer]

These insights raise important questions about the role that digital tools can play in assisting individual rehabilitation following stroke. In engaging with a portal, stroke survivors and carers might attempt to mitigate the negative impact of stroke on their lives, managing their vulnerability and the limitations of their condition.

### Hesitancy: Questioning the Benefits of Access to Personal Health Data

The role that digital technology can play in the management of health proved daunting for some individuals grappling with the effects of stroke. Some participants felt that digital technology needs to offer resources to encourage engagement, counteracting the hesitancy:

I think I’ve come to the conclusion that I’m too long in the tooth with my stroke. I’ve actually been through it, and I am quite happy to live with it. I haven’t got any of the anxieties, I’m not worried about my health on a daily basis, so I would need a portal to go and actually check things up.

[Jacob, stroke survivor]

The potential that the portal might add additional responsibilities and concerns was reflected upon by carers:

That graph that’s going upwards looks great because it’s suggesting Daniel is making some improvements, but again, if you are not making improvements...I don’t want to go in and populate it, and I don’t want to see that graph going down. Actually, yes, my dad could walk from the breakfast table into the living room to sit for the morning, and actually, now he can’t. I just think it’s kind of...for me, it’s a “decrepitness” score that would just be depressing, for him and me.

[Emma, carer]

Emma’s observations of the portal images relating to the Barthel Index score, a 10-item measure of activities of daily living [28], shown graphically on the self-living ability page, served as a reminder that in creating the profile for “Daniel,” the team had depicted a positive recovery trajectory. This also raised the possibility of a potential ceiling effect in the outcome measures used and displayed, in that they may not be sensitive enough to capture gains that are small yet still meaningful to the patient. Moreover, the design of the portal did not assist those with cognitive impairment and therefore placed an expectation and additional burden on the carer to complete in cases of severe disability following a stroke (Figure 3). Many stroke survivors and carers needed a clearer explanation of how the use of digital health technology would prove beneficial. Physical documents were preferred in offering straightforward access to the stroke survivor’s health information and saving additional work in having to ensure information was regularly updated online, crucial when considering caring responsibilities.

Hesitancy was also expressed among younger participants regarding the age of users, with concerns about who the portal was being designed for and how it would help them connect with other young stroke survivors. The portal was not designed to enable social media–type features, but participants under the age of 67 felt that this would be an important element in making the portal successful in engaging and offering relevant and purposeful information for younger stroke survivors, providing a valuable service that other groups sometimes lacked:

I know that it’s good to join in these kinds of groups so that you can talk about it and stuff like that, but I just find that people are just complaining, not trying to find solutions. And then last time, when I attended [charity group], they were talking about “Oh, these professionals, they don’t really tell you what to do” and stuff like that, and I mean, like, just purely out of trying to help, I just said, “You can call them and stuff like that, you can ask for information, or you can just do your own research,” and then they said, “Oh, we’ve got no time for that.” I was, like, “Okay. All right. Maybe I should just shut up.” [laughs] I mean, some of them were, like, my age, or in their twenties, so I’m quite surprised at how negative they can be, but I do understand, a stroke is not an easy thing.

[Miriam, stroke survivor]

### Support: Sharing Knowledge of Stroke

Many stroke survivors stated that they had sought wider professional and peer support to assist in their recovery. For stroke survivors, portal technologies are a potential platform on which to build connections, enabling individuals to gain knowledge and share experiences of their condition:

It’s about creating a community, so feeling like you’re part of a community and that the community can help. So it may even be that rather than, “Are there questions you need answers to?” it’s, “Is there advice you might give someone that we haven’t covered as well?” that you might want on there, so that people are feeling they can be creating a positive input, it’s not just always negative, that “I need this answer to this question so I’ve come here.”

[Caroline, stroke survivor]

For Caroline, “creating a community,” or a platform through. which she could seek educational resources, would allow her to answer questions in an environment in which she felt supported and understood. In establishing an environment in which stroke survivors could ask and respond to questions, Caroline felt that patient portals could potentially provide collective support by enabling the “creation of a positive” space in what can often feel like a negative environment poststroke.

While we did not raise the possibility of the portal offering connections to educational resources or peers, this suggestion arose when we asked if there was anything else that we might need to consider in the portal design:

You didn’t ask to be a carer, but you are one, and you do it because you love who you are looking after. “Here’s how we now recommend wiping a bottom or cleaning a catheter.” That stuff is largely missing for me. And sometimes I get it right, and I’m very pleased with myself...but sometimes I’m conscious that I could be causing more trouble.

[Emma, carer]

A patient portal could potentially ensure that reliable information is available. Awareness of stroke consequences was mentioned as being essential in enabling informal carers, friends, and family to assist in recovery. Participants noted that the portal might aid in reducing feelings of loneliness and isolation experienced by stroke survivors. Digital technologies could act as an intermediary between individuals and their wider networks in communicating the realities of their condition:

Somewhere where they can actually begin to understand exactly how disruptive a stroke is, it might make them feel a little bit more connected…I think just trying to explain to family what they could do to help, because sometimes just a phone call, and a phone call with some sort of understanding of what we’re going through, so the content can be modified by them to ask “How’s your hand working?” or “Have you tried this?” rather than the rather stilted conversations one has—“How are you, where have you been, what’s work like?”—all those sort of things.

[John, carer]

Through inclusion and use of patient portals, stroke survivors and carers could create positive, safe, and caring environments in which health problems relating to stroke can be considered. More importantly, they can ensure that their health needs are communicated among family, friends, or individuals who care for them as they learn to adjust to and navigate life poststroke.

## Discussion

This study examines the complex social dynamics between digital health engagement and information provision, challenging common perceptions that digital tools are simple, favorable solutions for managing chronic illnesses such as stroke. The tension between enablement and burden is evident within our study and reflects broader processes of responsibility within health care [29]. The portal appeared to offer people greater autonomy over their health through knowledge and management of their condition. This was evident in the cases of Hannah and Miriam, who viewed the portal as facilitating a process by which individuals “set their mind to recovery.” Moreover, this digital intervention appeared to offer opportunities to assist individuals like James in the self-management of his health poststroke. In reflecting on the “unmet” long-term needs of stroke survivors [30], our study suggests that digital interventions could prove essential in information provision for young and midlife stroke survivors who are more likely to fall outside the follow-up system [31]. More notably, the promotion of remote and self-managed recovery may serve to support psychological well-being, which stroke survivors feel is often considered secondary to physical health [32-35].

For carers like Gemma, the portal’s potential to offer an overview of her partner Chris’s health could prove reassuring in the long-term management of stroke recovery. However, it is important to note that the portal appears to simultaneously transfer responsibility for health monitoring from clinical services to individuals and their families. Navigating the care system can pose a series of challenges for individuals, with many people feeling unclear regarding resources and opportunities for support available to them, particularly given that stroke survivors (and relatives/carers) often have complex needs [4,6]. Therefore, while “passive information provision” has been associated with higher levels of anxiety for stroke survivors, “active information provision” has resulted in lower levels of anxiety and depression among carers [36]. Emma’s concern about tracking abilities in activities of daily living becoming a “decrepitness score” and negatively impacting stroke survivors and carers raises important questions about how digital health can add “data collectors” to the long list of tasks completed by carers; this, in turn, raises questions about who truly benefits from such technologies. Emma reminds us of the challenges that caregivers face in negotiating time, competence, and responsibility in managing their care recipient’s needs [37-39]. Furthermore, her interview reflects the pressures placed on caregivers to “advocate for stroke survivors” in ensuring that the stroke survivors’ interests are put first in all health-related engagements [40].

Our findings reveal how digital health technologies can inadvertently reproduce social inequalities. This is evident through accounts of young stroke survivors like Miriam, who struggled to find relevant peer support, while older participants faced technological barriers. This highlights the impacts of stroke-specific factors, such as cognitive impairment, aphasia, and varying recovery trajectories, creating additional layers of exclusion reinforced by ethnic, social, and gender inequalities. The emphasis participants placed on community connection challenges the individualistic focus of most digital health interventions. This can be seen in participants’ accounts, like those of Maria, a stroke survivor, and John, a carer, who felt isolated because of the disconnect between their lived experience of stroke and their family members’ limited understanding of the impact of stroke.

As such, portals designed for individual self-management may be less important than those facilitating social awareness and support. Resonating with the work of Denham et al. [38], participant accounts stress the need to provide an “honest” and realistic understanding of life after stroke, recognizing that effective digital health tools must address relational as well as medical needs.

### Strengths and Limitations

This study has both strengths and limitations. One strength is that participants were diverse in terms of time since stroke, ethnicity, and age. Another is that the study highlights the potential of digital interventions in enabling individuals to make informed decisions in relation to their health through access to information about their condition. Moreover, many participants noted the importance of such interventions in recovery, with the portal offering the potential to support stroke survivors and their wider network of support. While we attempted to engage stroke survivors experiencing aphasia, our engagement strategy was limited to interviews that were mostly held online, depending on geographical location, with many participants based in London or the south of England. Good technological ability or high levels of assistance from stroke survivors’ carers were also needed for participants to engage in online interviews. Moreover, we did not engage participants who were not fluent in the English language or who were experiencing severe, disabling complications because of their stroke. Participants might have benefited from the opportunity to directly interact with the portal, rather than being shown a series of photographs to illustrate its function. This decision was made because the portal required further development before undertaking usability and feasibility trials, which will be held at a later stage.

### Conclusions

This study raised several questions about who the portal was for, the purpose it served, and who was excluded from using digital technologies. For stroke survivors and carers, the promise of empowerment through digital self-management must be balanced against recognition of the social complexity of chronic illness experiences. Effective portal design requires attention given not only to people’s medical needs but also to the relational contexts and time frame in which recovery occurs. Participants recognized the potential benefits that a patient portal might offer, but further research is needed to determine the effective use of portals over extended periods across various populations.

## Supplementary material

10.2196/89787 Multimedia Appendix 1 Outline of portal features in prototype outline.

10.2196/89787 Multimedia Appendix 2 Stroke survivor interview topic guide.

## Abbreviations

PPI: Patient and Public Involvement
SF-12: 12-item Short-Form survey
SLSR: South London Stroke Register
