# Supplementary material for: Anticipated Effects of Using a Poststroke Patient Portal for Stroke Survivors and Carers: Semistructured Interview Study
Source: J Med Internet Res. 2026 Nov 11;28:e89787. doi: 10.2196/89787 (PMC13754254; doi:10.2196/89787)
Supplement: Supplementary file 1 [file jmir-v28-e89787-s001.docx]

Appendix 1: Outline of Portal Features in Prototype Outline
