# Supplementary material for: Anticipated Effects of Using a Poststroke Patient Portal for Stroke Survivors and Carers: Semistructured Interview Study
Source: J Med Internet Res. 2026 Nov 11;28:e89787. doi: 10.2196/89787 (PMC13754254; doi:10.2196/89787)
Supplement: Supplementary file 2 [file jmir-v28-e89787-s002.docx]

**Appendix 2**

Stroke Survivor Interview Topic Guide

**Introduction to the project**

This study explores the information needed by stroke survivors, carers, clinicians, researchers, healthcare managers and policy makers to help improve stroke care. Findings from this study will help direct the design and contents of a patient-friendly portal (a secure, web-based application that will provide stroke survivors and their carers access to personalised information about stroke and recovery. In the first instance, and in our trial in South London, this information will primarily come from the research data we collect on stroke survivors and their strokes, but it could potentially link to other data too. We will also look to collect information via the portal, through questionnaires, and wearable devices from stroke survivors, to contribute to future research.

1. **Can you please tell me a little about yourself?**

*Prompts***:**

When did you have your stroke?

What work did you do before your stroke? What about now?

How does your stroke affect you now?

Do you live alone? Who do you live with?

Does anyone help you in your everyday life because of the impact of your stroke?

How old are you?

So we can make sure we’re speaking to a diverse group of stroke survivors, please can you tell me how you would describe your ethnicity?

2. **What information is important to you in allowing you to manage your health post-stroke?**

*Prompts*

What about in relation to:

- Treatments?
- hospital appointments, test results and other services?

3. **How do you currently manage information relating to your health?**

P*rompts*

Do you use any tools to help manage this information?

Do you receive help in managing this information? If so, from who?

4. **Were you asked by nurse/doctor/specialist/health professional to monitor/record aspects of your health condition?**

*Prompts*

What aspects?

How did you do this? Did you use any tools? (electronic tools like websites, apps or even paper and pen)

5. **Do you currently access your health records online or have you sought to in the past?**

Prompts:

If not, is this something you’d be interested in doing? Why?

If so, what did you do? (Repeat prescriptions, check data etc).

6. **Have you used technology (e.g. NHS app/GP surgery app) to help manage your health in any way?**

*Prompts*

If yes:

- What did you use the apps for?
- What worked well with this App?
- What worked less well? How might that be improved?
- What was your lasting impression of the technology?
- Would you use it again?

If no:

- What prevent you from using this form of technology?
- What would encourage you to consider up taking technology to assist in the management of your health information?

7. **How would having access to your health information make you feel (e.g., encouraged, supportive, nervous or anxious)?**

Prompts:

How concerned are you with privacy and security with regards to your health information when using a patient portal?

The next half of the interview will focus on the design of the portal, and how we can make this work for you as a stroke survivor. We are interested in your thoughts and opinions on what element of your health in relation to your stroke are important to you.

8. **What springs to mind when you think of a portal?** (show images of portal to participant)

We’re thinking of things like Smart Metres, and apps that allow you to control your central heating. Even down to fitness Apps and wearable technology, like Apple Health and Fit Bits.

9. **What do you think is most important when making a portal user-friendly?**

*Prompts*

What would prevent/make you hesitant about using a patient portal? How could we overcome this problem?

Where would you want to access your information? Phone/Tablet/Computer?

Does your stroke affect your ability to use a portal? In what ways? Is there anything we could do to make it easier for you to use?

10. **What do you consider a priority for the portal to include for stroke survivors?**

*Prompts*

Why is this important to you? (If appropriate) How will this aid your recovery/experience post-stroke?

11. **What would you not like to see in a patient portal for stroke survivors? Why?**
12. **What would be important to you as a stroke survivor to ensure your continued engagement with the portal beyond initial use?**

*Prompts*

Health information? Monitoring health condition(s)? Getting information about stroke? Getting information about how their stroke fits into wider strokes?

13. **How do you think the portal could benefit you as a stroke survivor?**
14. **Do you have anything else that you would like to add that you feel as a stroke survivors may prove beneficial to us when considering the design of the portal?**

Would you be interested in apps/website which allow you to monitor and assess your health on a regular basis?
